# Cohort Profile: the Million Women Study

**DOI:** 10.1093/ije/dyy065

**Published:** 2018-06-04

**Authors:** Jane Green, Gillian K Reeves, Sarah Floud, Isobel Barnes, Benjamin J Cairns, Toral Gathani, Kirstin Pirie, Siân Sweetland, TienYu Owen Yang, Valerie Beral, Million Women Study Collaborators, Emily Banks, Valerie Beral, Lucy Carpenter, Carol Dezateux, Jane Green, Julietta Patnick, Richard Peto, Valerie Beral, Gillian Reeves, Sarah Floud, Jane Green, Hayley Abbiss, Simon Abbott, Rupert Alison, Krys Baker, Angela Balkwill, Isobel Barnes, Valerie Beral, Judith Black, Roger Blanks, Kathryn Bradbury, Anna Brown, Benjamin Cairns, Andrew Chadwick, Dave Ewart, Sarah Ewart, Sarah Floud, Toral Gathani, Laura Gerrard, Adrian Goodill, Jane Green, Lynden Guiver, Alicia Heath, Carol Hermon, Darren Hogg, Isobel Lingard, Sau Wan Kan, Tim Key, Nicky Langston, Kath Moser, Kirstin Pirie, Alison Price, Gillian Reeves, Keith Shaw, Emma Sherman, Rachel Simpson, Helena Strange, Siân Sweetland, Sarah Tipper, Ruth Travis, Lyndsey Trickett, Anthony Webster, Clare Wotton, F Lucy Wright, Tienyu Owen Yang, Heather Young

**Affiliations:** Cancer Epidemiology Unit, Nuffield Department of Population Health, University of Oxford, Oxford, UK

## Why was the cohort set up?

The Million Women Study started recruiting participants over 20 years ago, in 1996. The initial stimulus was to obtain robust prospective information on the risk of breast cancer associated with use of different types of menopausal hormone therapy (HT). When planning the necessary large-scale prospective study, an equally important aim was to obtain reliable information on the effects of other potentially modifiable factors that affect women’s health as they age.

In the early 1990s use of HT increased rapidly in the UK and elsewhere, stimulated in part by claims that use of HT could improve general well-being and increase life expectancy. By the mid-1990s, however, worldwide evidence was beginning to show that HT preparations increased breast cancer risk, though there was little information about the effect of the type of HT most commonly used in Europe, containing both oestrogens and progestagens.^1^ It was also clear that women born in the 1940s, who reached adulthood in the 1960s, had considerably different lifestyles compared with previous generations. For example, large proportions had begun smoking and using oral contraceptives as teenagers and young adults, and the long-term effects of these behaviours could not be studied reliably until the 1990s. At the same time there was growing concern about the effects of the increasing prevalence of obesity, and claims that other factors such as diet had important effects on health, all of which required large-scale prospective evidence.

The UK National Health Service (NHS) provides extraordinarily efficient ways of establishing and maintaining long-term follow-up for large prospective epidemiological studies. Over 99% of the UK population, and all Million Women Study participants, are registered with the NHS, and every individual has a unique NHS number. Electronic linkage, using each individual’s NHS number, to routinely collected NHS databases provides virtually complete follow-up information about deaths, emigrations, cancer registrations and hospital admissions.

The NHS Breast Screening Programme invites all UK women registered with the NHS, of a specified age, for free routine breast screening every 3 years. In 1996–2001 the programme routinely invited women aged 50–64 years for mammographic screening, by sending each individual a letter offering them a specific date and time at a specific screening centre. In 66 NHS screening centres, the Million Women Study recruitment questionnaire was included with the invitation letter for screening. Pilot studies in 1994–96 had shown that inclusion of a questionnaire with the invitation did not affect uptake of breast screening.^2^

The coordinating centre for the Million Women Study is based in the Cancer Epidemiology Unit, Nuffield Department of Population Health, University of Oxford. The study was set up in collaboration with the NHS Breast Screening Programme, and is now funded mainly by the UK Medical Research Council and Cancer Research UK. Further information, including the study protocol, copies of questionnaires, data collected, data access policy and list of publications can be found on the study website [www.millionwomenstudy.org].

## Who is in the cohort?

**Figure 1:**
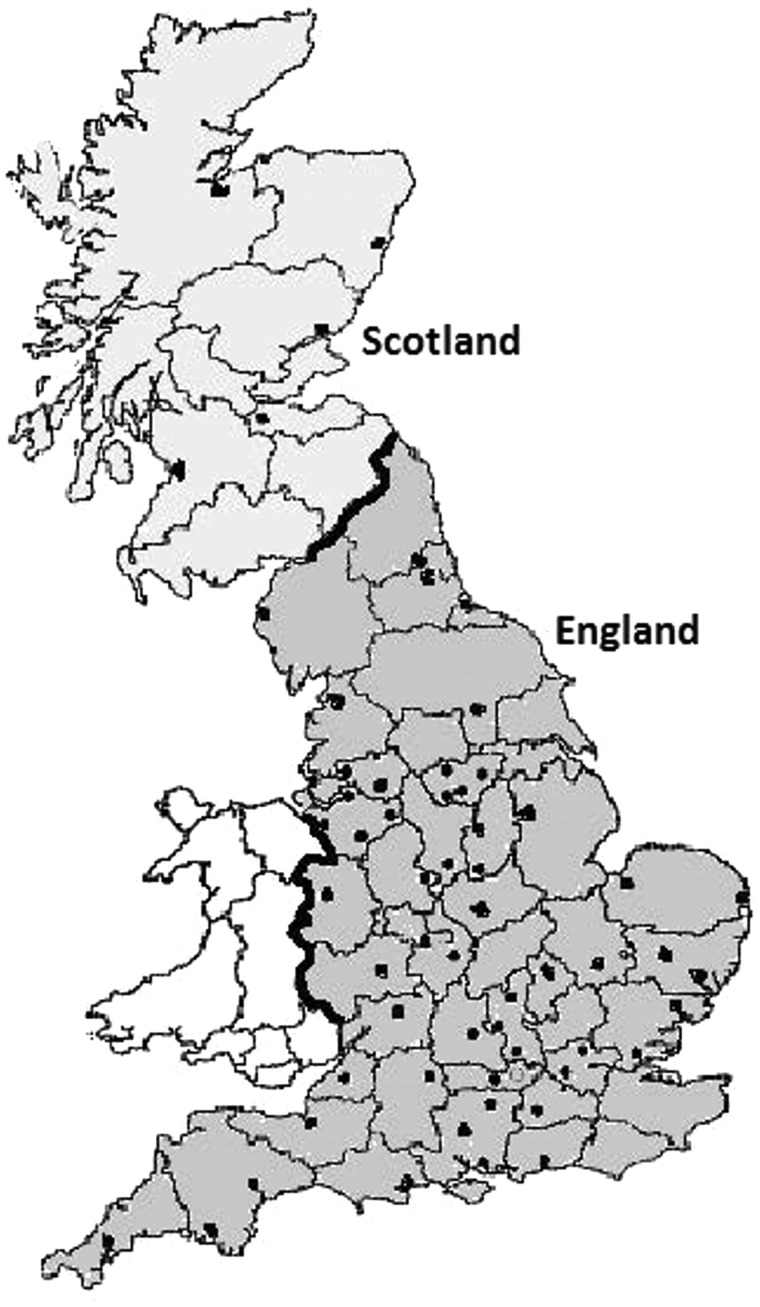
Location of the 66 NHS breast screening clinics through which Million Women Study participants were recruited ▪. Figure adapted from Figure 1 of the original article ‘The Million Women Study Collaborative Group: The Million Women Study: design and characteristics of the study population [peer-reviewed research]. *Breast Cancer Res* 1999; **1**: 73–80. The original article is an open access article distributed under the terms of the Creative Commons Attribution License [http://creativecommons.org/licenses/by/2.0], which permits unrestricted use, distribution, and reproduction in any medium, provided the original work is properly cited.

Between 1996 and 2001, the Million Women Study recruited about one in every four UK women born in 1935–1950, i.e. in the eligible age range (50–64 years) at the time of recruitment. The 66 NHS breast screening centres that recruited participants (Figure 1) covered about half of the UK population. Half of the women invited by the participating screening centres brought completed questionnaires with them when they were screened, or posted their questionnaires to the Million Women Study Coordinating Centre. The recruitment questionnaire asked about sociodemographic, anthropometric, behavioural and reproductive factors, and also about women’s past health. Participants gave written consent for re-contact and for follow-up through screening clinic and other medical records.

The study design and methods were first reported in 1999.^3^ Selected characteristics of the cohort at recruitment, and details of subsequent follow-up, are shown in Table 1. The cohort included 1.32 million women without previous cancer. The median year of birth of the cohort was 1942, and at recruitment in 1996–2001 women were aged 56 [standard deviation (SD) 5] years on average. The study includes women with a wide range of backgrounds, behaviours and lifestyles at recruitment and their characteristics were, not surprisingly, broadly similar to those of all UK women of their age at the time. A fifth were current smokers at recruitment. Typical of UK women of their age, almost a quarter reported that they did not drink alcohol, and among the drinkers the average consumption was about five drinks per week. The cohort includes some 150 000 women (11%) from the lowest quintile of the national deprivation index, based on the Townsend score,^4^ and so although the proportion is somewhat less than the national average, there are sufficiently large numbers to study reliably associations across the full range of socioeconomic status in the UK.

**Table 1.** The Million Women Study: selected characteristics of the 1.32 million women at recruitment, and details of follow-up

Characteristics at recruitment	
Number of women	1 319 475
Year of birth, median (IQR)	1942 (1938-46)
Age at recruitment, years, mean (SD)	56 (5)
Nulliparous	11%
Number of children, in parous women, mean (SD)	2 (1)
Ever use of oral contraceptives	59%
Current smoker	20%
Does not drink alcohol	24%
Alcohol, drinks/week (in drinkers), mean (SD)	5 (5)
Height, cm, mean (SD)	162 (7)
Weight, kg, mean (SD)	69 (13)
Body mass index, kg/m^2^, mean (SD)	26 (5)
Any physical activity, two or more times per week	55%
Strenuous physical activity, two or more times per week	21%
Menopausal hormone therapy (HT), current use	33%
Follow-up	
To 1 April 2017	
Deaths	14% (185 233)
Total number of hospital admissions	7 631 806
Women with at least one hospital admission with:	
Any diagnosis	85% (1 128 056)
Ischaemic heart disease	13% (172 485)
Stroke	4% (46 481)
Fracture	10% (137 654)
Lost to follow-up	1% (19 705)
To 1 January 2016	
Women with incident cancer:	
All sites (excluding non-melanoma skin cancer)	15% (201 988)
Breast cancer	5% (70 305)
Colorectal cancer	2% (22 203)
Lung cancer	2% (21 482)

Percentages shown are among women with available information.

## How often have they been followed up?

The Million Women Study is an open-ended prospective study of women in England and Scotland. The entire cohort is followed up annually by record linkage to routinely collected NHS data on deaths, emigrations, cancers and hospital admissions. For some study participants, additional electronic linked health data are available, for example for primary care consultations and prescriptions, and for cancer screening. Table 2 gives a summary of the type of health and health care follow-up data routinely collected, and of data providers.

**Table 2.** The Million Women Study: summary of electronic linkages to routinely collected NHS health and healthcare data

	Data provider	Population with data	Dates	Data
Deaths	England: ONS/NHS Digital	Entire cohort	Annual update	ICD-10 cause of death
	Scotland: ISDScotland			
Emigrations and other loss to follow-up	England: ONS/NHS Digital	Entire cohort	Annual update	Reasons and dates
	Scotland: ISDScotland			
Cancer registrations	England: ONS/NHS Digital	Entire cohort	Annual update	ICD-10 cancer site
	Scotland: ISDScotland			ICD-O tumour morphology
Hospital inpatient/day patient admissions	England: HES/NHS Digital	Entire cohort	Annual update	ICD-10 diagnoses
	Scotland: ISDScotland			OPCS4 procedures
Cancer screening	Public Health England	Participants in England	Ad hoc, latest to 2013	Dates of invitations for breast, cervix and bowel screening and attendance
Cancer outcomes and services dataset	Public Health England	Participants in England	Ad hoc, latest to 2017	Tumour characteristics (e.g. stage, grade)
Primary care	England: Clinical Practice Research Datalink	100 000 women in England	Ad hoc, latest to 2013	Diagnosis and prescribing (Read/OXMIS codes)
	Scotland: ISDScotland	100 000 women in Scotland	Ad hoc, latest 2009-15	Dispensing of drugs prescribed in primary care

NHS Digital (formerly the Health and Social Care Information Centre): [https://digital.nhs.uk/].

ISDScotland = Information Services Division Scotland (NHS National Services Scotland): [https://www.isdscotland.org/].

ONS = Office for National Statistics: [https://www.ons.gov.uk/]. HES = Hospital Episode Statistics.

Data on deaths and hospital admissions are available to 1 April 2017. By that date, 14% (185 233) of women had died, and 85% (1 128 056) had had at least one admission to hospital. Only 1.4% (*n* = 19 705) of the cohort had been lost to follow-up by emigration, withdrawal from the NHS or for some other reason. Such women are included in relevant analyses until the date of their withdrawal from follow-up. Data on registered cancers have been provided to 1 January 2016, and 15% (*n* = 201 988) of women had an incident cancer (excluding non-melanoma skin cancer) registered by this date.

Thus far, four re-survey postal questionnaires (at 3, 8, 12 and 15 years, on average, after recruitment) have been sent to all survivors, to obtain information on important factors that may change over time, such as smoking, alcohol consumption, weight and physical activity, and to collect new information on other exposures. Information has also been collected for subsets of participants through additional postal and online questionnaires, e.g. for diet and daily activities.

## What has been measured?

At recruitment, and through the subsequent four re-survey questionnaires, information on about 1400 variables has been collected, about a range of sociodemographic, lifestyle and other personal factors. The questionnaires, and a summary table of the information collected at each, can be viewed on the study website. Some characteristics of women who responded to the 3-year and 8-year re-surveys are shown in Table 3.

**Table 3.** Million Women Study: selected characteristics of study participants recorded at re-survey

	3-year re-survey	8-year re-survey
Number returning a study questionnaire	837 985	661 583
Year completed, median (IQR)	2001 (2000–2003)	2006 (2006–2006)
Years between recruitment and re-survey, mean	3.3	7.8
Age, years, mean (SD)	60 (5)	64 (5)
Current smoker	12%	9%
Non-drinkers (<one drink of alcohol per week)	38%	37%
Alcohol, drinks/week (in drinkers), mean (SD)	7 (6)	8 (6)
Weight, kg, mean (SD)	69 (12)	70 (13)
Body mass index, kg/m^2^, mean (SD)	26 (5)	26 (5)
Menopausal hormone therapy (HT), current use	28%	8%
Good or excellent self-rated health	76%	80%
Happy usually or most of the time	83%	85%
Married or living with partner	80%	77%
Housework, hours per week, mean (SD)	15 (11)	n/a
Walking, hours per week, mean (SD)	5 (6)	n/a
Sleep, hours per day, mean (SD)	7 (1)	7 (2)
Plumper than average when 10 years old	15%	15%
Fruit consumption, 3+ servings per day	13%	19%
Salad or raw vegetables, 3+ servings per day	6%	7%
Cooked vegetables, 3+ servings per day	13%	16%
Never eat meat	3%	3%
Never eat fish	3%	2%
Eat no red or processed meat	13%	15%
Took aspirin during most of the past month	10%	17%
Took paracetamol during most of the past month	17%	27%
Took ibuprofen during most of the past month	9%	10%

Percentages shown are among women with available information.

One drink  =  approximately 10 g alcohol.

n/a =  not available (question not asked).

Various validation and other studies have been done in subsets of the cohort to address methodological issues of measurement error, regression dilution and changes in exposures over time.^5–10^ For example, height, weight, waist circumference, hip circumference and blood pressure were measured for about 4000 women to quantify measurement errors in self-reported data.^5^ Self-reported information on menopausal HT use,^6^ cervical screening,^7^ and knee and hip replacement^8^ has been compared with that in NHS records. Online 24-h recall of diet has been collected and repeat dietary questionnaires completed, to assess the repeatability of self-reported dietary data over time.^9^^,^^10^

Clinical outcomes recorded in routinely collected NHS data have been compared with those recorded in medical notes, primary care records or screening records for breast cancer,^11^ vascular disease,^12^ motor neurone disease^13^ and dementia.^14^ All the investigations have indicated the excellent reliability of the routinely collected NHS diagnostic data. Since 2006, blood samples have been collected from about a 5% sample of women in the study for genetic and biochemical analyses, mainly concerning breast cancer.^15^^,^^16^

## What has it found? Key findings and publications

Full details of the wide range of findings and publications using Million Women Study data are available on the study website. Two key findings are as follows.

**Figure 2:**
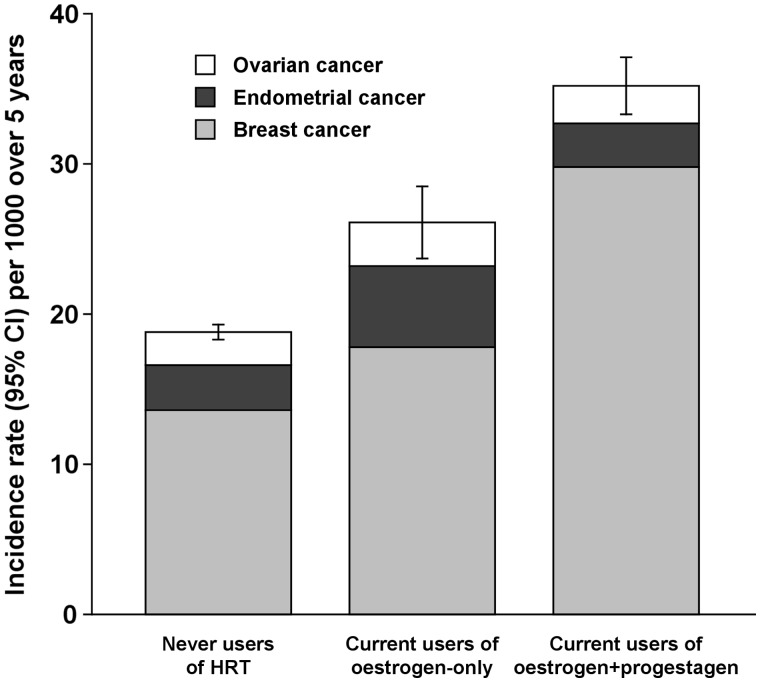
Million Women Study: incidence of cancers of the breast, ovary and endometrium in relation to use of menopausal hormone therapy (HRT). *Figure reprinted and adapted from Lancet* 2007;**369:**1703–10: Beral *et al.*, Ovarian cancer and hormone replacement therapy in the Million Women Study. Copyright (2007), with permission from Elsevier.

**Figure 3:**
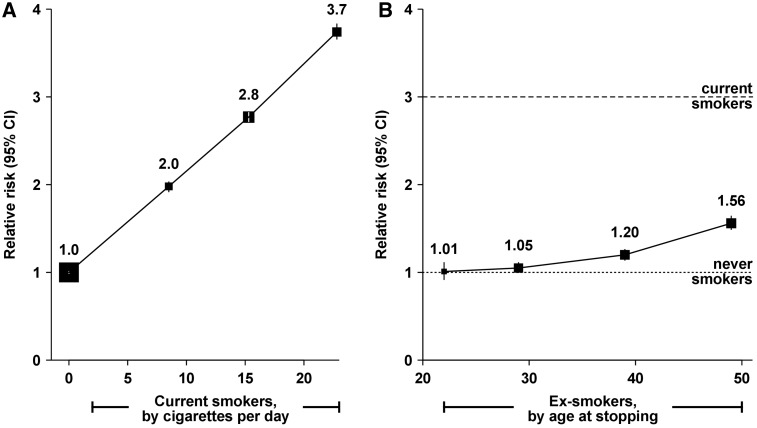
All-cause mortality in current and in ex-smokers in the Million Women Study. Adapted with permission from Pirie *et al.*, *Lancet* 2013:**381:**133–41.

- We have shown that the risk of cancers of the breast and endometrium vary substantially by the type of HT used.^17^^,^^18^ Use of oestrogen-progestagen preparations causes much greater increases in the risk of breast cancer than oestrogen-only preparations, whereas the reverse is found for endometrial cancer. For ovarian cancer, use of HT slightly increases risk, but there is no difference in the effects of oestrogen-progestagen and oestrogen-only preparations.^19^ Because breast cancer is much more common than endometrial or ovarian cancer, the overall effect of HT on the three cancer types is dominated by the effects on breast cancer. Hence users of oestrogen-progestagen HT have substantially higher absolute risks of the three cancers together than users of oestrogen-only preparations or than women who do not use HT (Figure 2).
- We have shown that the hazards of smoking, and also the benefits of stopping smoking, in women are greater than previously thought.^20^ The Million Women Study is well placed to estimate these risks, because women born in the 1930s and 1940s were the first generation in the UK to start smoking substantial numbers of cigarettes regularly in early adulthood and to continue to do so throughout their lives. Smokers were three times more likely to die prematurely than never smokers, the equivalent of losing 11 years of life, on average. We also found that stopping smoking is more effective in reducing the excess risk than previously thought; a woman who stops smoking at age 40 avoids about 90% of the excess risk associated with continued smoking (Figure 3).

## What are the main strengths and weaknesses of the study?

Strengths include: the large size of the cohort, which provides sufficient statistical power to study outcomes reliably and to compare risks across disease types and subtypes; the prospective collection of exposure information; and the virtually complete, long-term follow-up for major outcomes through linkage to routinely collected, complete, reliable, national electronic health records. Re-surveys and measurements of various factors provide repeated measures of changing exposures, so that analyses can take account of measurement error, regression dilution and changes in exposure over time. Data linkage offers the potential for continued, cost-effective follow-up for many more years and, as routine health databases expand, for incorporation of additional data. The study has the advantage that data are available to address a wide range of questions, now and in the future. Its observational nature, however, means that it is sometimes difficult to establish causation.

The great majority of study participants are of White ethnicity (96%) and the cohort has no information on men. Blood samples are available only for a minority of participants, and were provided several years after recruitment. The current lack of routinely recorded information available for NHS outpatient diagnoses, and the fact that information is currently available from primary care only for a sample of the cohort, mean that there is at present limited information on outcomes not involving hospital admission.

## Can I get hold of the data? Can I find out more?

The Million Women Study welcomes proposals for data access and sharing from bona fide researchers; details of the study, the data available and the data access application process can be found on the study website [http://www.millionwomenstudy.org/data_access]. Enquiries should be directed through the website to the Administrator, Richard Doll Centenary Archive.

**Profile in a nutshell**

- The Million Women Study is a population-based prospective study of 1.3 million UK women, recruited in 1996-2001, when the women were aged 56 years on average. It includes about one in every four UK women born in 1935-50, i.e. in the eligible age range (50-64 years) at the time of recruitment.
- Information on a wide range of sociodemographic and behavioural factors has been collected at recruitment, at four subsequent re-surveys and by online questionnaire.
- Follow-up through electronic record linkage to routinely collected NHS data on deaths, emigrations, cancer registrations and hospital admissions is virtually complete.
- After almost 20 years follow-up, only 1% have been lost to follow-up, 14% have died, 15% have had an incident cancer registration and 85% have had at least one hospital admission.
- The Million Women Study uses a controlled data access model [http://www.millionwomenstudy.org/data_access].

## Funding

Main funding is from Cancer Research UK and the UK Medical Research Council.

### Collaborators

The Million Women Study Advisory Committee: Emily Banks, Valerie Beral, Lucy Carpenter, Carol Dezateux (Chair), Jane Green, Julietta Patnick, Richard Peto, Cathie Sudlow. Co-principal investigators: Valerie Beral, Gillian Reeves, Sarah Floud, Jane Green. Current Million Women Study Co-ordinating Centre staff: Hayley Abbiss, Simon Abbott, Rupert Alison, Krys Baker, Angela Balkwill, Isobel Barnes, Valerie Beral, Judith Black, Roger Blanks, Kathryn Bradbury, Anna Brown, Benjamin Cairns, Andrew Chadwick, Dave Ewart, Sarah Ewart, Sarah Floud, Toral Gathani, Laura Gerrard, Adrian Goodill, Jane Green, Lynden Guiver, Alicia Heath, Carol Hermon, Darren Hogg, Isobel Lingard, Sau Wan Kan, Tim Key, Nicky Langston, Kath Moser, Kirstin Pirie, Alison Price, Gillian Reeves, Keith Shaw, Emma Sherman, Rachel Simpson, Helena Strange, Siân Sweetland, Sarah Tipper, Ruth Travis, Lyndsey Trickett, Anthony Webster, Clare Wotton, F Lucy Wright, Tienyu Owen Yang, Heather Young. The following NHS Breast Screening Centres took part in the recruitment and breast screening follow-up for the Million Women Study: Avon, Aylesbury, Barnsley, Basingstoke, Bedfordshire and Hertfordshire, Cambridge and Huntingdon, Chelmsford and Colchester, Chester, Cornwall, Crewe, Cumbria, Doncaster, Dorset, East Berkshire, East Cheshire, East Devon, East of Scotland, East Suffolk, East Sussex, Gateshead, Gloucestershire, Great Yarmouth, Hereford and Worcester, Kent, Kings Lynn, Leicestershire, Liverpool, Manchester, Milton Keynes, Newcastle, North Birmingham, North East Scotland, North Lancashire, North Middlesex, North Nottingham, North of Scotland, North Tees, North Yorkshire, Nottingham, Oxford, Portsmouth, Rotherham, Sheffield, Shropshire, Somerset, South Birmingham, South East Scotland, South East Staffordshire, South Derbyshire, South Essex, South Lancashire, South West Scotland, Surrey, Warrington Halton St Helens and Knowsley, Warwickshire Solihull and Coventry, West Berkshire, West Devon, West London, West Suffolk, West Sussex, Wiltshire, Winchester, Wirral, Wycombe.

## References

[1] Collaborative Group on Hormonal Factors in Breast Cancer. Breast cancer and hormone replacement therapy: collaborative reanalysis of data from 51 epidemiological studies of 52 705 women with breast cancer and 108 411 women without breast cancer. Lancet 1997;350:1047–59. 10213546

[2] Banks E, Richardson A, Beral V et al Effect on attendance at breast cancer screening of adding a self-administered questionnaire to the usual invitation to breast screening in Southern England. J Epidemiol Community Health 1998;52:116–19. 9578859 10.1136/jech.52.2.116 PMC1756668

[3] Banks E, Beral V, Reeves G; on behalf of the Million Women Study Collaborative Group. The Million Women Study: design and characteristics of the study population. Breast Cancer Res 1999;1:73–80. 11056681 10.1186/bcr16 PMC13913

[4] Townsend P, Phillimore P, Beattie A. Health and Deprivation: Inequality and the North. Beckenham, UK: Croom Helm, 1988.

[5] Wright L. Validity over time of self-reported anthropometric variables during follow-up of a large cohort of UK women. BMC Med Res Methodol 2015;15:81. 26450616 10.1186/s12874-015-0075-1 PMC4599695

[6] Banks E, Beral V, Cameron R et al Agreement between general practice prescription data and self-reported use of hormone replacement therapy and treatment for various illnesses. J Epidemiol Biostat 2001;6:357–63. 12036270 10.1080/13595220152601837

[7] Canfell K, Beral V, Green J, Cameron R, Baker K, Brown A. The agreement between self-reported cervical smear abnormalities and screening programme records. J Med Screen 2006;13:72–75. 16792828 10.1258/096914106777589687

[8] Liu B, Sweetland S, Beral V, Green J, Balkwill A, Casabonne D; Million Women Study Collaborators. Self-reported information on joint replacement and cholecystectomy agrees well with that in medical records. J Clin Epidemiol 2007;60:1190–94. 17938062 10.1016/j.jclinepi.2007.02.007

[9] Liu B, Young H, Crowe FL et al Development and evaluation of the Oxford WebQ, a low-cost, web-based method for assessment of previous 24 h dietary intakes in large-scale prospective studies. Public Health Nutr 2011;14:1998–2005. 21729481 10.1017/S1368980011000942

[10] Roddam AW, Spencer E, Banks E et al Reproducibility of a short semi-quantitative food group questionnaire and its performance in estimating nutrient intake compared with a 7-day diet diary in the Million Women Study. Public Health Nutr 2005;8:201–13. 15877913 10.1079/phn2004676

[11] Gathani T, Bull D, Green J, Reeves G, Beral V; Million Women Study Collaborators. Breast cancer histological classification: agreement between the Office for National Statistics and the National Health Service Breast Screening Programme. Breast Cancer Res 2005;7:R1090–96. 16457689 10.1186/bcr1352 PMC1410775

[12] Wright FL, Green J, Canoy D, Cairns BJ, Balkwill A, Beral V. Vascular disease in women: comparison of diagnoses in hospital episode statistics and general practice records in England. BMC Med Res Methodol 2012;12:161. 23110714 10.1186/1471-2288-12-161 PMC3514155

[13] Doyle P, Brown A, Beral V, Reeves G, Green J. Incidence of and risk factors for motor neurone disease in UK women: a prospective study. BMC Neurol 2012;12:25. 22559076 10.1186/1471-2377-12-25 PMC3512483

[14] Brown A, Kirichek O, Balkwill A et al Comparison of dementia recorded in routinely collected hospital admission data in England with dementia recorded in primary care. Emerg Themes Epidemiol 2016;13:11. 27800007 10.1186/s12982-016-0053-z PMC5084368

[15] Travis RC, Reeves GK, Green J et al Gene-environment interactions in 7610 women with breast cancer: prospective evidence from the Million Women Study. Lancet 2010;375:2143–51. 20605201 10.1016/S0140-6736(10)60636-8 PMC2890858

[16] Reeves GK, Travis RC, Green J et al Incidence of breast cancer and its subtypes in relation to individual and multiple low-penetrance genetic susceptibility loci. JAMA 2010;304:426–34. 20664043 10.1001/jama.2010.1042

[17] Beral V, Banks E, Reeves G, Bull D; on behalf of the Million Women Study Collaborators. Breast cancer and hormone-replacement therapy in the Million Women Study. Lancet 2003;362:419–27. 12927427 10.1016/s0140-6736(03)14065-2

[18] Beral V, Bull D, Reeves G; for the Million Women Study Collaborators. Endometrial cancer and hormone-replacement therapy in the Million Women Study. Lancet 2005;365:1543–51. 15866308 10.1016/S0140-6736(05)66455-0

[19] Beral V, Bull D, Green J, Reeves G; on behalf of the Million Women Study Collaborators. Ovarian cancer and hormone replacement therapy in the Million Women Study. Lancet 2007;369:1703–10. 17512855 10.1016/S0140-6736(07)60534-0

[20] Pirie K, Peto R, Reeves GK, Green J, Beral V; for the Million Women Study Collaborators. The 21st century hazards of smoking and benefits of stopping: a prospective study of one million women in the UK. Lancet 2013;381:133–41. 23107252 10.1016/S0140-6736(12)61720-6 PMC3547248

